# Overlap of Pain and Depression Among Nursing Home Residents With Advanced Alzheimer’s Disease and Related Dementia

**DOI:** 10.1093/geroni/igaa057.138

**Published:** 2020-12-16

**Authors:** Emmanuelle Belanger, Richard Jones, Gary Epstein-Lubow, Kate Lapane

**Affiliations:** 1 Brown University School of Public Health, Providence, Rhode Island, United States; 2 Brown University, Providence, Rhode Island, United States; 3 UMass Medical School, Worcester, Massachusetts, United States

## Abstract

Physical and psychological suffering are interrelated and should be assessed together as part of palliative care delivery. We aimed to describe the overlap of pain and depressive symptoms among long-stay nursing home (NH) residents with advanced Alzheimer’s disease and related dementia (ADRD), and to determine the incidence of pain and depressive symptoms. We conducted a retrospective study of a US national sample of fee-for-Service Medicare beneficiaries who became long-stay NH residents in 2014-2015, had two consecutive quarterly Minimum Dataset assessments (90 and 180 days +/- 30 days), and had a diagnosis of ADRD in the Chronic Condition Warehouse and moderate to severe cognitive impairment (N= 92,682). We used descriptive statistics and Poisson regression models to examine the incidence of each symptom controlling for age, sex, and concurrent hospice care. Sub-groups with self-reported and observer-rated symptoms (pain/PHQ-9) were modelled separately, as were those switching between the two. The prevalence of depressive symptoms was low (5.7%), while pain was more common (18.2%). Across various subgroups, 2% to 4% had both pain and depression, but between 20% and 25% were treated with both antidepressants and scheduled analgesia. Depressed residents at baseline had an incidence rate ratio (IRR) of pain of 1.2 at the second assessment, while the residents with pain at baseline had an IRR of depressive symptoms of 1.3 at the second assessment. Our results support the expected relationship between pain and depressive symptoms in a national sample of long-stay NH residents with advanced ADRD, suggesting the need for simultaneous clinical management.

